# Impact of changes of positive end-expiratory pressure on functional residual capacity at low tidal volume ventilation during general anesthesia

**DOI:** 10.1007/s00540-012-1411-9

**Published:** 2012-05-15

**Authors:** Daizoh Satoh, Shin Kurosawa, Wakaba Kirino, Toshihiro Wagatsuma, Yutaka Ejima, Akiko Yoshida, Hiroaki Toyama, Kei Nagaya

**Affiliations:** 1 Department of Anesthesiology and Perioperative Medicine, Tohoku University Postgraduate Medical School, 1-1 Seiryomachi, Aoba-ku, Sendai, Miyagi 980-8574 Japan; 2 Department of Anesthesia, Tohoku Kosei Nenkin Hospital, Sendai, Japan

**Keywords:** Functional residual capacity, Positive end-expiratory pressure, Low tidal volume ventilation, General anesthesia

## Abstract

**Purpose:**

Several reports in the literature have described the effects of positive end-expiratory pressure (PEEP) level upon functional residual capacity (FRC) in ventilated patients during general anesthesia. This study compares FRC in mechanically low tidal volume ventilation with different PEEP levels during upper abdominal surgery.

**Methods:**

Before induction of anesthesia (awake) for nine patients with upper abdominal surgery, a tight-seal facemask was applied with 2 cmH_2_O pressure support ventilation and 100 % O_2_ during FRC measurements conducted on patients in a supine position. After tracheal intubation, lungs were ventilated with bilevel airway pressure with a volume guarantee (7 ml/kg predicted body weight) and with an inspired oxygen fraction (FIO_2_) of 0.4. PEEP levels of 0, 5, and 10 cmH_2_O were used. Each level of 5 and 10 cmH_2_O PEEP was maintained for 2 h. FRC was measured at each PEEP level.

**Results:**

FRC awake was significantly higher than that at PEEP 0 cmH_2_O (*P* < 0.01). FRC at PEEP 0 cmH_2_O was significantly lower than that at 10 cmH_2_O (*P* < 0.01). PaO_2_/FIO_2_ awake was significantly higher than that for PEEP 0 cmH_2_O (*P* < 0.01). PaO_2_/FIO_2_ at PEEP 0 cmH_2_O was significantly lower than that for PEEP 5 cmH_2_O or PEEP 10 cmH_2_O (*P* < 0.01). Furthermore, PEEP 0 cmH_2_O, PEEP 5 cmH_2_O after 2 h, and PEEP 10 cmH_2_O after 2 h were correlated with FRC (*R* = 0.671, *P* < 0.01) and PaO_2_/FIO_2_ (*R* = 0.642, *P* < 0.01).

**Conclusions:**

Results suggest that PEEP at 10 cmH_2_O is necessary to maintain lung function if low tidal volume ventilation is used during upper abdominal surgery.

## Introduction

Pulmonary gas exchange and respiratory mechanics are usually impaired during general anesthesia and muscle paralysis. Functional residual capacity (FRC) decreases approximately 20 % during induction of anesthesia [1]. Intraabdominal surgery might aggravate FRC loss. Reduced FRC can be restored somewhat through ventilation using positive end-expiratory pressure (PEEP). Nevertheless, few reports in the literature describe studies of the influence of PEEP level upon FRC in ventilated patients during administration of general anesthesia. FRC was reduced by induction of anesthesia, but no report in the literature has described a comparison of FRC values with those of stepwise increase of PEEP during upper abdominal surgery. We measured FRC for mechanically ventilated general anesthesia with different PEEP levels during upper abdominal surgery and subsequently compared them with awake levels measured for the subject in the supine position. We hypothesized that FRC, compliance, and arterial oxygenation show positive effects of increased PEEP, and that 5 cmH_2_O PEEP or 10 cmH_2_O PEEP increased FRC nearly to the awake level. Different techniques have been developed and evaluated recently for FRC measurements during mechanical ventilation. The wash-in or wash-out of a tracer gas in a multiple breath maneuver is apparently best applied at bedside. Promising techniques for nitrogen or oxygen wash-in/wash-out with reasonable accuracy and repeatability have been presented [2]. This technique, which estimates FRC with good precision using a change of FIO_2_ of only 0.1, has been evaluated in volunteers and in patients with acute respiratory distress syndrome [3].

## Materials and methods

We studied nine patients with American Society of Anesthesiologists Physical Status Classification System grade 1 or grade 2 who had been scheduled for elective upper abdominal surgery. Informed consent was obtained from each patient. The study was approved by the Tohoku University Hospital Ethics Committee. No patient had cardiac or pulmonary disease. The arterial line, inserted under local anesthesia, was used for the study protocol. Monitoring included invasive arterial blood pressure, a continuous electrocardiogram, and peripheral oxygen saturation (SpO_2_). After induction of anesthesia and tracheal intubation, end-tidal carbon dioxide concentration (ETCO_2_), airway pressure, tidal volume, and respiratory rate were monitored and recorded.

### Anesthesia

**Fig. 1:**
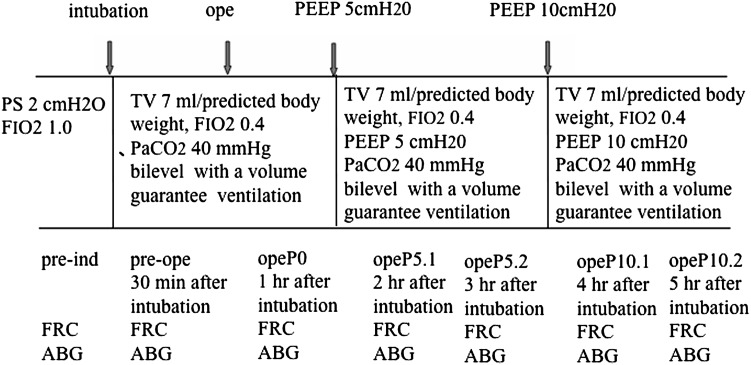
Schematic protocol used for this study. *PS* pressure support ventilation, *FRC* functional residual capacity, *ABG* arterial blood gases, *TV* tidal volume, *PEEP* positive end-expiratory pressure. Predicted body weight: men, 49.9 + 0.91 [height (cm), 152.4]; women, 45.4 + 0.91 [height (cm), 152.4]

No premedication was administered. Before induction of anesthesia (Awake), a tight-seal facemask was used for spontaneous ventilation (Engström Carestation; GE Healthcare, Madison, WI, USA). The facemask was applied with 2 cmH_2_O pressure support ventilation using an inspired oxygen fraction (FIO_2_) of 1.0 during FRC measurement (FRC INview). The FRC was designated as the facemask FRC value minus facemask dead space (100 ml). Arterial blood gas samples were collected from subjects who were in a supine position (Fig. 1). Patients were ventilated with 2 cmH_2_O pressure support ventilation at awake time. Anesthesia was induced as target-controlled infusion of propofol 4–5 μg/ml with a calculated target plasma, fentanyl 1–2 μg/kg i.v., and rocuronium bromide 0.6 mg/kg i.v.

Predicted body weight was calculated as 45.4 + 0.91 × [height (cm) − 152.4] for women or 49.9 + 0.91 × [height (cm) − 152.4] for men. After tracheal intubation, anesthesia was maintained with a propofol target plasma drug concentration of 2.5–3 μg/ml and remifentanil 0.2–0.4 μg/kg/min with thoracic epidural analgesia. A neuromuscular block was monitored continuously using a nerve stimulator. Rocuronium bromide was infused as necessary.

Lungs were ventilated in bilevel airway pressure with volume guarantee (pressure-regulated volume control). Upper airway pressure was maintained for 1.3 s and with FIO_2_ 0.4. Ventilation was started. The tidal volume of 7 ml/kg was estimated according to the predicted body weight. The respiratory rate was adjusted to maintain ETCO_2_ at 35–40 mmHg. FRC measurements and arterial blood gas samples were taken from 30 min after intubation (pre-ope). From 1 h after intubation, FRC measurements and arterial blood gas samples were obtained during upper abdominal surgery (opeP0). The abdominal cavity was opened for opeP0.

Subsequent ventilations started with PEEP 5 cmH_2_O. After 1 and 2 h PEEP 5 cmH_2_O, FRC measurements and arterial blood gas samples were collected (opeP5.1 and opeP5.2). FRC measurements and arterial blood gas samples were also collected at 1 and 2 h after the stepwise increase of PEEP 10 cmH_2_O (opeP10.1 and opeP10.2).

The quasistatic compliance of the respiratory system was calculated as the tidal volume divided by the inspiratory plateau pressure minus the end-expiratory pressure. Values are expressed as mean ± SD. Comparison among groups was performed using two-way analysis of variance for repeated one-way measurements. Correlation between PEEP and FRC or the PaO_2_/FIO_2_ ratio was analyzed using Pearson’s correlation. For all comparisons, *P* < 0.05 was considered significant.

## Results

Figure 1 presents patient data of the nine patients studied.

**Fig. 2:**
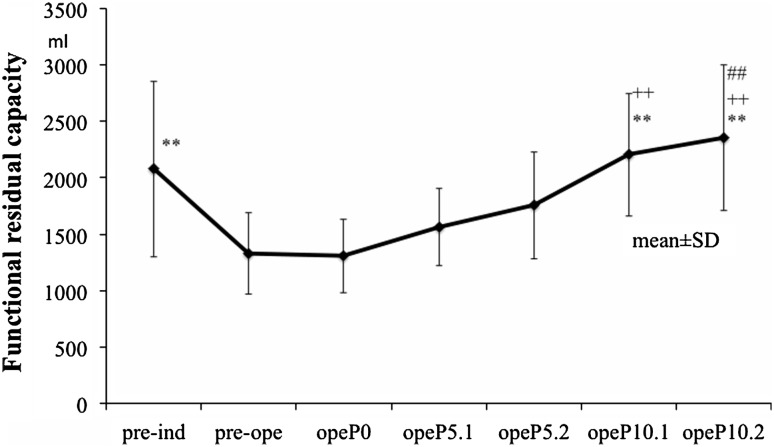
Functional residual capacity for PEEP levels studied. **P < 0.01 versus preoperative (pre-op) and opeP0, ^++^ *P* < 0.01 versus opeP5.1, ^##^ *P* < 0.01 versus opeP5.2 pre-induction (pre-ind). *P* postoperative, *FRC* measured pre-ind FRC − mask dead space (100 ml)

Figure 2 shows data obtained for FRC. The value of FRC awake was significantly higher than that for pre-ope or opeP0 (*P* < 0.01). The FRC ope10.1 and ope10.2 values were significantly higher than that for opeP5.1 (*P* < 0.01). The FRC for opeP10.2 was significantly higher than that for ope5.2 (*P* < 0.01).

**Fig. 3:**
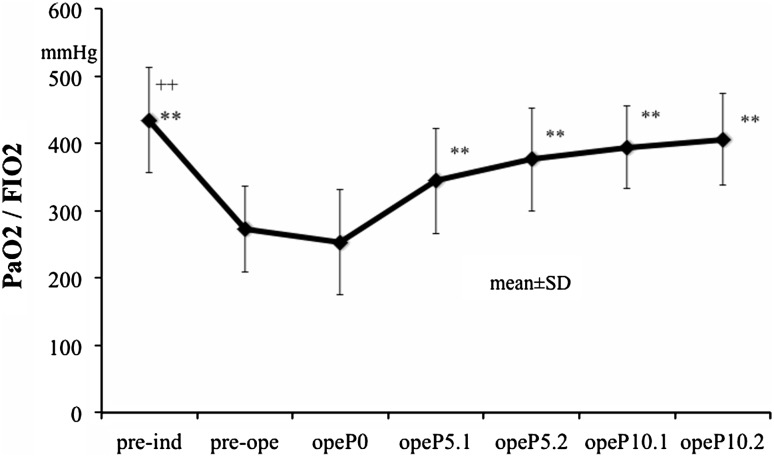
PaO_2_/FIO_2_ for PEEP levels studied. **P < 0.01 versus pre-op and opeP0, ^++^ *P* < 0.01 versus opeP5.1

Figure 3 presents oxygenation data. The PaO_2_/FIO_2_ awake was significantly higher than that for pre-ope, opeP0, or ope5.1 (*P* < 0.01). PEEP 5 cmH_2_O and PEEP 10 cmH_2_O were significantly higher than either pre-ope or opeP0 (*P* < 0.01) (Table 1).

**Table 1:** Patient characteristics (*n* = 9)

Gender (male:female)	5:4
Age (years)	69 ± 7
Weight (kg)	64 ± 8
Height (cm)	158 ± 6
Predicted body weight (kg)	53 ± 6
Body mass index (BMI)	26 ± 43
Pancreaticoduodectomy	4
Pancreatectomy	2
Hepatic lobectomy	2
Reconstructive operation of biliary tract	1

Values are mean ± SD

### Correlation

**Fig. 4:**
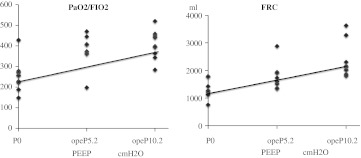
PEEP levels at opeP0, opeP5.2, and opeP10.2 were correlated with FRC (*R* = 0.671, *P* < 0.01) and PaO_2_/FIO_2_ (*R* = 0.642, *P* < 0.01)

PEEP levels at opeP0, opeP5.2, and opeP10.2 were correlated with FRC (*R* = 0.671, *P* < 0.01) and PaO_2_/FIO_2_ (*R* = 0.642, *P* < 0.01) (Fig. 4).

### Quasistatic compliance (Table 2)

Compliance was significantly higher for opeP10.2 than for opeP5.1 (*P* < 0.01).

**Table 2:** Respiratory mechanics and hemodynamic data

	Pre-ope	opeP0	opeP5.1	opeP5.2	opeP10.1	opeP10.2
PIP (cmH_2_O)	12 ± 2	13 ± 2	17 ± 2*^,#^	16 ± 2*^,#^	21 ± 2*^,#,§,‡^	20 ± 2*^,#,§,‡^
PaCO_2_ (mmHg)	44 ± 5	44 ± 6	43 ± 6	42 ± 6	43 ± 6	43 ± 6
Compliance(ml/cmH_2_O)	36 ± 10	35 ± 9	35 ± 9	37 ± 9	38 ± 9	41 ± 10^#,§^
MAP (mmHg)	98 ± 12	114 ± 9*	117 ± 22*	109 ± 14	110 ± 19	113 ± 14
Heart rate (bpm)	63 ± 10	72 ± 16	75 ± 16*	76 ± 15*	82 ± 17*	81 ± 17*

Data are expressed as mean ± SD

Quasistatic compliance = tidal volume/(inspiratory plateau pressure − PEEP)

*ope* operation, *P* postoperative day, *PIP* peak inspiratory pressure, *MAP* maximum arterial pressure

* *P* < 0.01 versus pre-operative (pre-ope), ^#^ *P* < 0.01 versus opeP0, ^§^ *P* < 0.01 versus opeP5.1, ^‡^ *P* < 0.01 versus opeP5.2

### Hemodynamics (Table 2)

Maximum arterial pressure and heart rate were lowest at pre-ope. No significant difference was found in hemodynamics among different PEEP groups.

## Discussion

The first main finding of our study was that PEEP showed positive effects on FRC, compliance, and PaO_2_/FIO_2_ ratio, indicating that patients undergoing upper abdominal surgery might benefit from PEEP 10 cmH_2_O.

The FRC examinations were performed using an Engström Carestation (GE Healthcare) equipped with the FRC Inview monitoring feature. The FRC is determined using the change of lung nitrogen volume after a step change in the inspired oxygen fraction. In the Engström Carestation, when ventilated with FIO_2_ 1.0, where the O_2_ consumption was calculated from the CO_2_ production with a default RQ of 0.85, very high measurement precision was attained [2].

Results show that FRC was reduced by 37 % from the awake level after the induction of anesthesia. As previously reported, FRC is reduced by approximately 15 % during anesthesia induced with thiopentone and maintained with halothane at FIO_2_ 0.35 [4]. Our protocol was FIO_2_ 1.0 at induction of anesthesia, which engendered absorption atelectasis because of the high concentration of oxygen. The mechanisms underlying reduction of FRC include muscle paralysis, decreased chest wall recoil, increased abdominal pressure, atelectasis formation, and gas entrapment behind a closed airway [5]. Our results showed no significant change in FRC between that after induction of anesthesia and that during operation at zero end-expiratory pressure (ZEEP). During abdominal operation, FRC increased transiently when the cavity was opened [6]. However, during the surgery procedure, FRC decreased and returned to post-induction levels at the end of the procedure. Indeed, pulmonary gas exchange, which is impaired following induction of anesthesia, deteriorates during laparotomy but not during peripheral surgery. The difference is probably explained by the effect of surgical influences (packs, retractors, etc.) on FRC [1]. At opeP0, surgical influences (retractors) were started.

Results of this study demonstrated that PEEP 10 cmH_2_O reached nearly the same FRC level as that of awake FRC. No data exist to elucidate waking FRC for different PEEP levels, showing intraoperative FRC changes. Neumann et al. [7] presented measured mean FRC data for postoperative patients at different PEEP levels (0, 5, 10 cmH_2_O). Their study revealed that PEEP increased FRC at levels of PEEP 5 and 10 cmH_2_O; PEEP decreased FRC after reversion to PEEP of 0 cmH_2_O. Pelosi et al. [8] reported that PEEP 10 cmH_2_O did not improve respiratory function in paralyzed or anesthetized postoperative patients. Their mean PaO_2_/FIO_2_ ratio was 436 mmHg, and their tidal volume was 8–12 ml/kg ideal body weight. Our mean PaO_2_/FIO_2_ ratio was 253 mmHg, and tidal volume was 7 ml/kg ideal body weight at PEEP 0 cmH_2_O intraoperatively. Lower tidal volume might engender atelectasis, especially if PEEP is low or not used at all. Sufficient PEEP must be used to minimize atelectasis and to maintain oxygenation [9].

Determann et al. described a randomized controlled nonblinded preventive trial comparing mechanical ventilation with tidal volume of 10 versus 6 ml/kg in critically ill patients without acute lung injury (ALI) at the onset of mechanical ventilation. Mechanical presentation with 10 ml/kg is associated with sustained cytokine production in plasma [10]. Those results suggest that mechanical ventilation with conventional tidal volumes contributed to the development of lung injury in patients without ALI at the onset of mechanical ventilation. This theory applies to mechanical ventilation during surgery.

Administration of PEEP alone increased the normally aerated lung fraction, which combined with a reduction of poorly aerated lung tissue while atelectasis remained unchanged [10]. An earlier study of postoperative mechanically ventilated obese patients (mean body mass index, 51) after abdominal surgery showed that PEEP 10 cmH_2_O increased PaO_2_, respiratory compliance, and FRC [8]. PEEP at 10 cmH_2_O was sufficient to maintain substantial improvement of respiratory function. Reportedly, PEEP higher than 10 cmH_2_O is associated with marked derangement of hemodynamics [11]. Rothen et al. [12] reported that static compliance and the amount of atelectasis estimated using computed tomography (CT) did not change in parallel. Maisch et al. [13] described that compliance indicates an optimal level of PEEP after recruitment in anesthetized patients, reporting that optimal PEEP was 10 cmH_2_O because, at that pressure level, the highest compliance value in conjunction with the lowest dead space fraction revealed a maximum amount of effectively expanded alveoli. Our results demonstrated that the quasistatic compliance was significantly higher for opeP 10.2 than for opeP 5.1. PEEP 10 cmH_2_O did not produce the pre-operation level of PaO_2_/FIO_2_, probably because of a ventilation and circulation mismatch by mechanical ventilation or because recruitment maneuvers were not used. The increased intrapleural pressure caused by PEEP might also increase the risk of barotrauma and cause changes to cardiovascular dynamics. Two trials reported postoperative barotrauma in both PEEP and ZEEP [14, 15]. The event rate was zero in both groups in both trials. The Imberger G group calculated an effect estimate of cardiac complication. Their comparison revealed RR of 0.3 for the PEEP group, which was not statistically significant [16]. It remains unclear whether the increases in FRC and *P*/*F* ratio are attributable to the increase in PEEP level, or to a time-dependent effect of PEEP, or both. During a time study of 5 h operation, PaO_2_ showed constant values at much lower tidal volumes of 6 ml/kg and PEEP 10 cmH_2_O (21 patients were enrolled; 13 cases were upper abdominal surgeries with abdominal opening) [17]. Following coronary artery bypass grafting, significant reduction of P (A–a) O_2_ during positive pressure ventilation at 10 cmH_2_O PEEP was compared with 0 cmH_2_O PEEP and 5 cmH_2_O PEEP during 6 h [18]. These data show that pulmonary oxygenation might maintain constant values at equal PEEP levels.

As one limitation of our study, PEEP was applied in a stepwise increasing fashion. We have no data for *P*/*F* ratio and FRC on time-dependent effect of PEEP. However, the same PEEP level was maintained for 2 h. Two hours after changing PEEP, the FRC data were slightly higher than at 1 h after changing PEEP, but the values were not significantly different. The PEEP levels at opeP0, opeP5.2, and opeP10.2 were correlated with FRC and with PaO_2_/FIO_2_. Consequently, the PEEP increase was inferred to have engendered the FRC increase. The PEEP levels might be applied in a random sequence to mitigate this potential bias in the results.

## Conclusions

Data reported herein suggest that if low tidal volume ventilation is used during upper abdominal surgery, then PEEP 10 cmH_2_O is necessary to maintain lung function.
